# Sustained Effect of Hyaluronic Acid in Subcutaneous Administration to the Cochlear Spiral Ganglion

**DOI:** 10.1371/journal.pone.0153957

**Published:** 2016-04-21

**Authors:** Yozo Inagaki, Masato Fujioka, Sho Kanzaki, Kotaro Watanabe, Naoki Oishi, Go Itakura, Akimasa Yasuda, Shinsuke Shibata, Masaya Nakamura, Hirotaka James Okano, Hideyuki Okano, Kaoru Ogawa

**Affiliations:** 1 Department of Otolaryngology Head and Neck Surgery, Keio University, Shinjuku-ku, Tokyo, Japan; 2 Department of Orthopedics, School of Medicine, Keio University, Shinjuku-ku, Tokyo, Japan; 3 Department of Physiology, School of Medicine, Keio University, Shinjuku-ku, Tokyo, Japan; Osaka University Graduate School of Medicine, JAPAN

## Abstract

The spatiotemporal distribution of drugs in the inner ear cannot be precisely evaluated because of its small area and complex structure. In the present study, we used hyaluronic acid (HA)-dispersed luciferin to image transgenic mice and to determine the effect of HA on controlled drug delivery to the cochlea. GFAP-luc mice, which express luciferase in cochlear spiral ganglion cells, were subcutaneously administered HA-luciferin (HA-sc) or luciferin dissolved in saline (NS-sc) or intraperitoneally administered luciferin dissolved in saline (NS-ip). The bioluminescence of luciferin was monitored in vivo in real time. The peak time and half-life of fluorescence emission were significantly increased in HA-sc-treated mice compared with those in NS-sc- and NS-ip-treated mice; however, significant differences were not observed in peak photon counts. We detected differences in the pharmacokinetics of luciferin in the inner ear, including its sustained release, in the presence of HA. The results indicate the clinical potential of using HA for controlled drug delivery to the cochlea.

## Introduction

The inner ear is a minute organ surrounded by bones and comprises a tight junction that serves as a blood—inner ear barrier between the inner ear fluid and systemic circulation. These characteristics impede efficient drug delivery, creating an urgent need to develop a topical drug delivery system (DDS) that enables efficient transport and subsequent maintenance of high drug concentrations to a part of the inner ear. Moreover, researchers face daunting obstacles presented by the anatomical, histological, and structural limitations of the inner ear that make it technically difficult to continuously monitor the concentrations of topically administered drugs without disrupting normal physiological pharmacokinetics.

To overcome these problems, we used transgenic mice (GFAP promoter-luciferase [GFAP-Luc)] [1]) that express high levels of luciferase (driven by the promoter of the gene encoding glial fibrillary acid protein) in the spiral ganglion of the auditory end-organ (cochlea) of the inner ear to develop a real-time imaging system for measuring the delivery and distribution of a drug to the spiral ganglion [2]. The broad linear range of the quantitative luciferase assay is widely used to analyze reactions in vitro, and commercially available imaging systems are employed to detect luciferase activity in vivo. In particular, the long wavelength of the light emitted by the luciferase reaction passes through tissues, allowing the detection of luminescence that originates deep inside the body of a rodent [1, 3]. We found that GFAP-Luc transgenic mice expressed luciferase in peripheral glial cells of the auricular skin and the cochlear spiral ganglion in the head and neck areas. Moreover, we found that surgical excision of the auricle from the same mouse made it possible to monitor the delivery of luciferin to the cochlear spiral ganglion as well as its pharmacokinetics [2].

Hyaluronic acid (HA) is a type of mucoperiosteum that is a component of the major extracellular matrix of organisms, and HA is widely used as a biomaterial [[4]. Because HA is a natural compound, it has good biocompatibility; its applications in the fields of orthopedics [5] and ophthalmology [6] attest to its high safety. Further, HA is viscoelastic with high wettability. Changing the cross-linking of macromolecular HA increases its molecular weight and viscosity. Dispersing a drug with hyaluronan optimized for wettability and viscoelasticity provides a DDS that facilitates the sustained release of an appropriate amount of drug at a therapeutically sufficient rate [7]. Current research focuses on drug delivery to the inside of the joints affected by diseases such as arthrosis and rheumatoid arthritis as well as to lacrimal fluid in patients with dry eye [5, 6].

Our aim in this study was to develop a DDS to provide sustained release of a drug combined with HA to the cochlear spiral ganglion. We investigated the variation in inner-ear pharmacokinetics depending on the composition and method of administration of the sustained-release preparation, and we assessed the sustained-release effect of HA on the cochlea. Further, we determined whether we could accurately assess drug delivery by the DDS to the inner ear. Our results illustrated that the administration of a luciferin-HA conjugate to the back of a mouse significantly increased the time to reach the maximum luciferase concentration and significantly increased the half-life of luciferin in the cochlea compared with subcutaneous or intraperitoneal administration of a saline solution of luciferin. The results further revealed that in vivo imaging using the luciferase transgenic mouse affords facile assessment of drug delivery to the inner ear.

## Methods

### Mice

Transgenic GFAP-Luc mice (FVB/N background [1]) were obtained from Xenogen Corporation (Alameda, CA) and backcrossed with CD1 mice (ICR, SLC Japan) for 8 or 9 generations. GFAP-Luc mice harbor a firefly luciferase gene expression cassette that is regulated by a 12-kb sequence comprising the murine *Gfap* promoter and intron 2 of the gene encoding human β-globin2 [1]. Luciferin delivered to the inner ear of these mice is oxidized by luciferase expressed by luciferase-expressing cells in the cochlear nerve and spiral ganglion, and a camera was used to detect the emitted photons. All experiments were approved by and conducted in accordance with the Animal Care and Use Committee of Keio University (Permit Number 08020), which is in accordance with the Guide for the Care and Use of Laboratory Animals (National Institutes of Health, Bethesda, MD, USA). Wild-type (FVB/N) mice served as controls. Because GFAP-Luc mice express luciferase in their earlobes, we surgically removed their earlobes until immediately above the eardrum before performing the experiments.

### Luciferin preparations

d-luciferin (Summit Pharmaceuticals International Corporation) was dissolved in saline or 1% hyaluronan (Seikagaku Corporation) to a final concentration of 15 mg/ml and then filtered.

### In vivo imaging

An IVIS Spectrum system and a charge-coupled device—optical macroscopic imaging system (Xenogen, Alameda, CA) were used for spatiotemporal detection of the luciferase—luciferin reaction as described previously [8], [9]. Images were captured after drug administration with the field-of-view set to 10 cm and with an integration time of 5 min. All images were analyzed using Living Image software (Xenogen). The optical signal intensity was expressed as photon flux (photon count) in units of photons/s/cm^2^/steradian. Each image is displayed as a pseudo-colored photon-count image superimposed onto a grayscale anatomical image. To quantify the emitted light, we defined regions of interest over the temporal bone and examined all values in the same region of interest. The photon counts between 5 and 290 min after administration were subjected to statistical analysis using the following method.

We divided GFAP-Luc mice into the following groups: 1) luciferin + saline (100 μl) intraperitoneal injection group (NS-ip: n = 7); 2) luciferin + saline (100 μl) subcutaneous injection group (NS-sc: n = 7); 3) luciferin + 1% HA (molecular weight = 2.9 × 10^6^) (100 μl) subcutaneous injection group (HA-sc: n = 7); and 4) Control, luciferin + saline (100 μl) subcutaneous injection group (wild-type: n = 2). We performed real-time quantitation of light emission (photon counts) using the IVIS Spectrum imaging system (Xenogen) to measure the pharmacokinetics of luciferin in the inner ear.

### Statistical analysis

We performed one-way analysis of variance of the data for the peak counts, peak times, half-lives, and total photon counts in the NS-ip, NS-sc, HA-sc groups, and wild-type groups. The half-life was defined as the time at which the emission time reached ≤50% of the initial peak value during the acquisition of photon counts. Significant differences among the 3 groups were analyzed using the Tukey method. Before conducting analysis of variance and the *t*-test to compare individual groups, we performed an F-test to evaluate the size of the experiments. All scores were averaged and analyzed using SPSS software 19.0 (IBM Corp, Armonk, NY).

## Results

**Fig 1:**
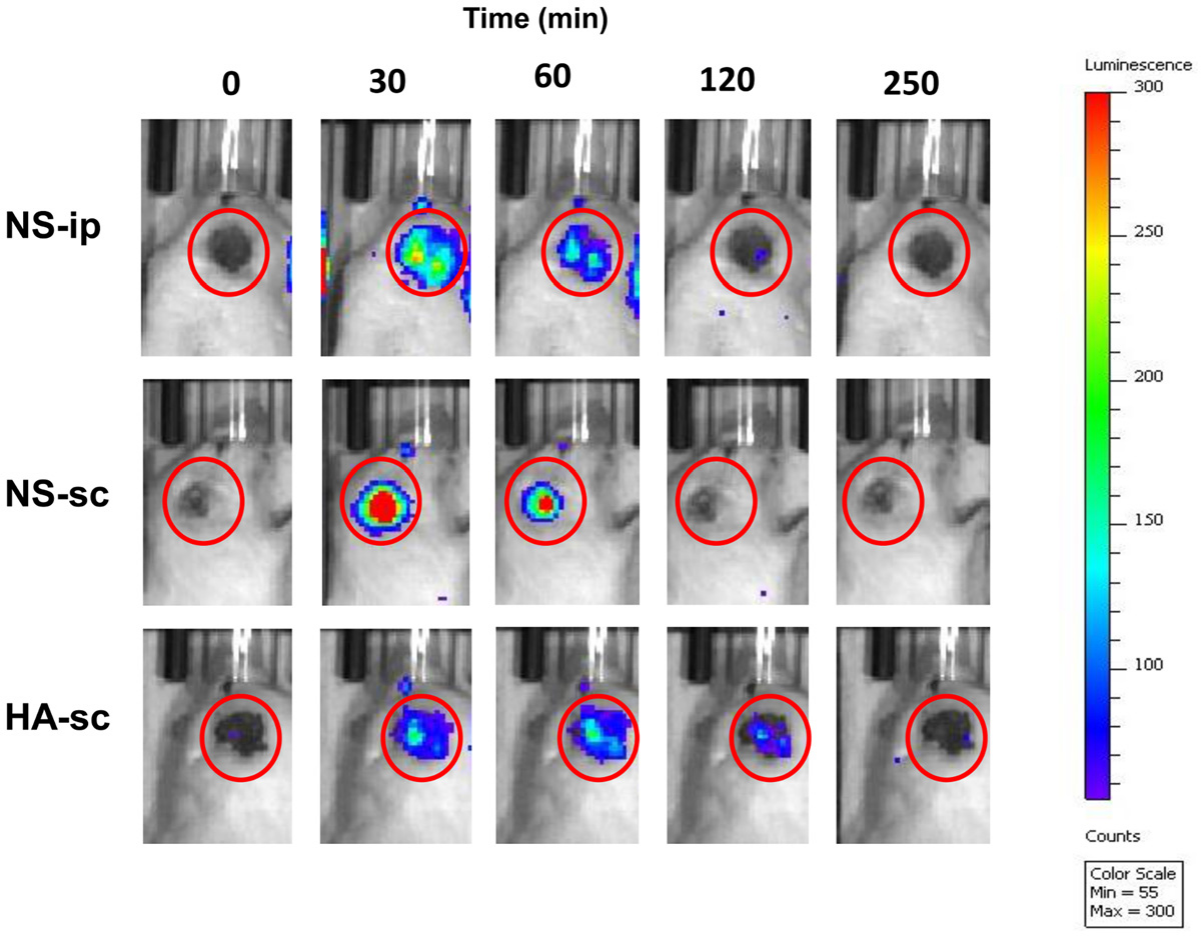
Representative images of individual mice administered luciferin. NS-ip: Luciferin + saline (100 μl) intraperitoneal injection group. NS-sc: Luciferin + saline (100 μl) subcutaneous injection group. HA-sc: Luciferin + 1% HA (molecular weight = 2.9 million) subcutaneous injection group.

**Fig 2:**
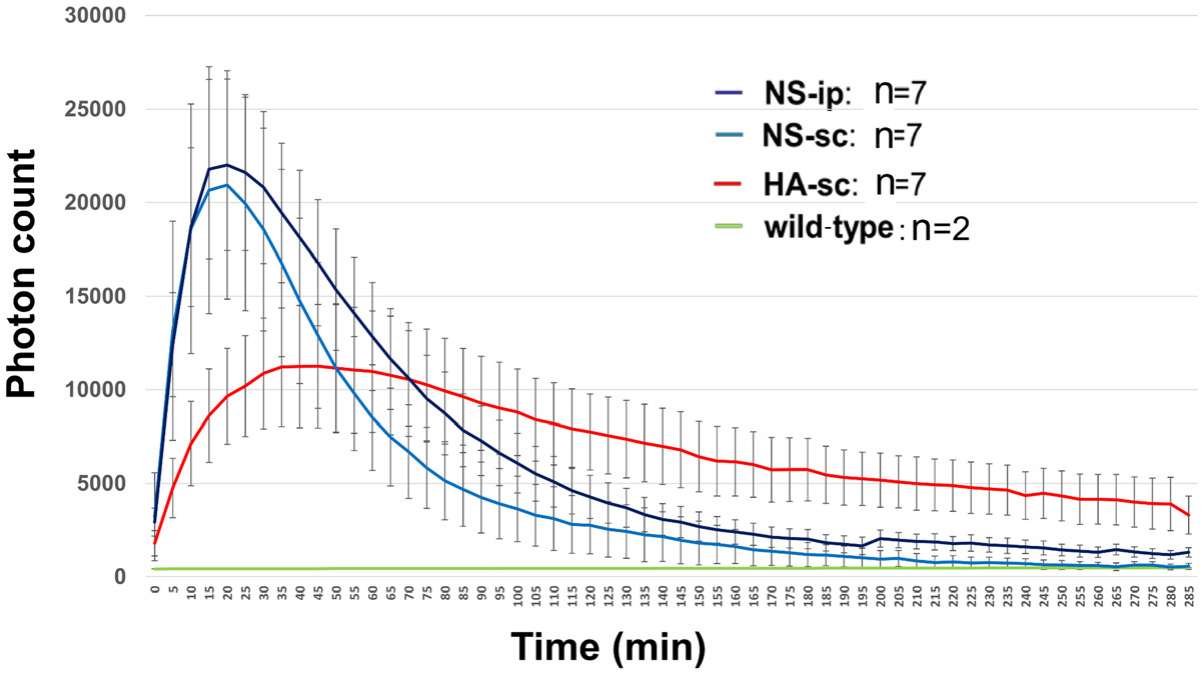
Time-dependent changes of photon counts of transgenic and wild-type mice administered luciferin via different routes. The designations of the groups are shown in Fig 1.

**Fig 3:**
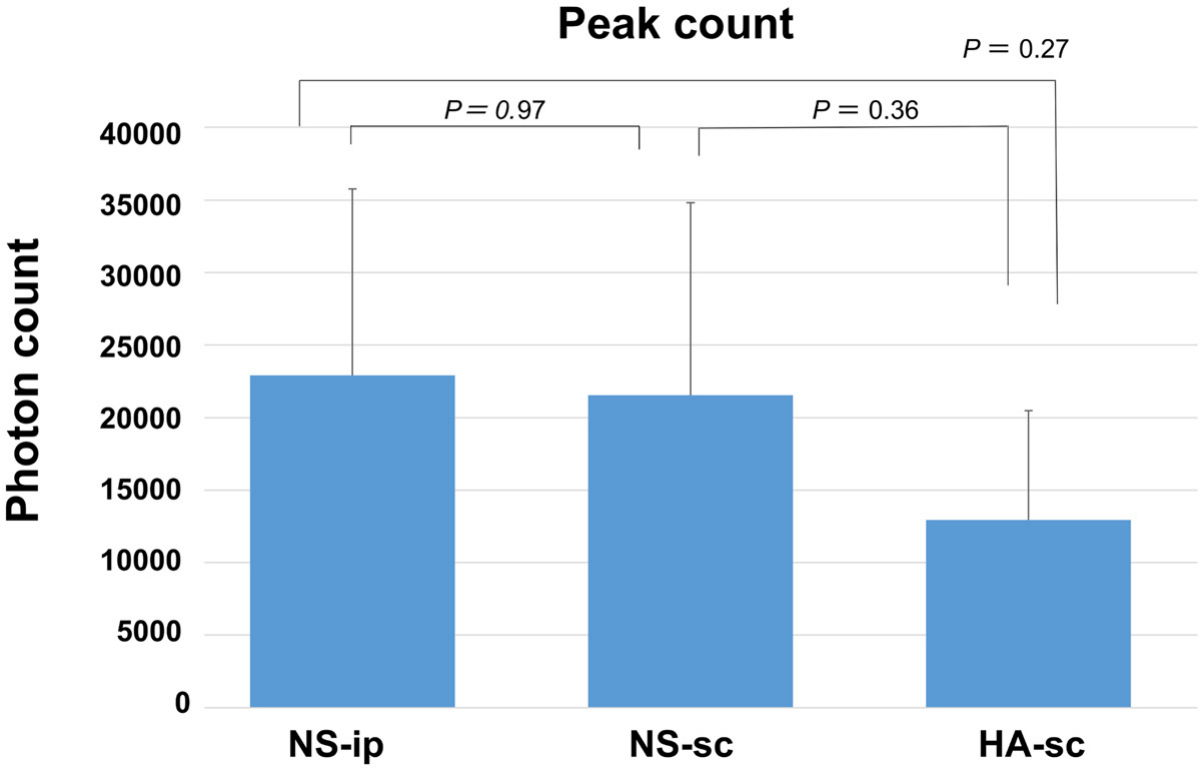
Peak photon count of each group of mice that were administered luciferin. The bar graph shows the average value of 7 mice in each group, and the error bars indicate the standard deviation.

**Fig 4:**
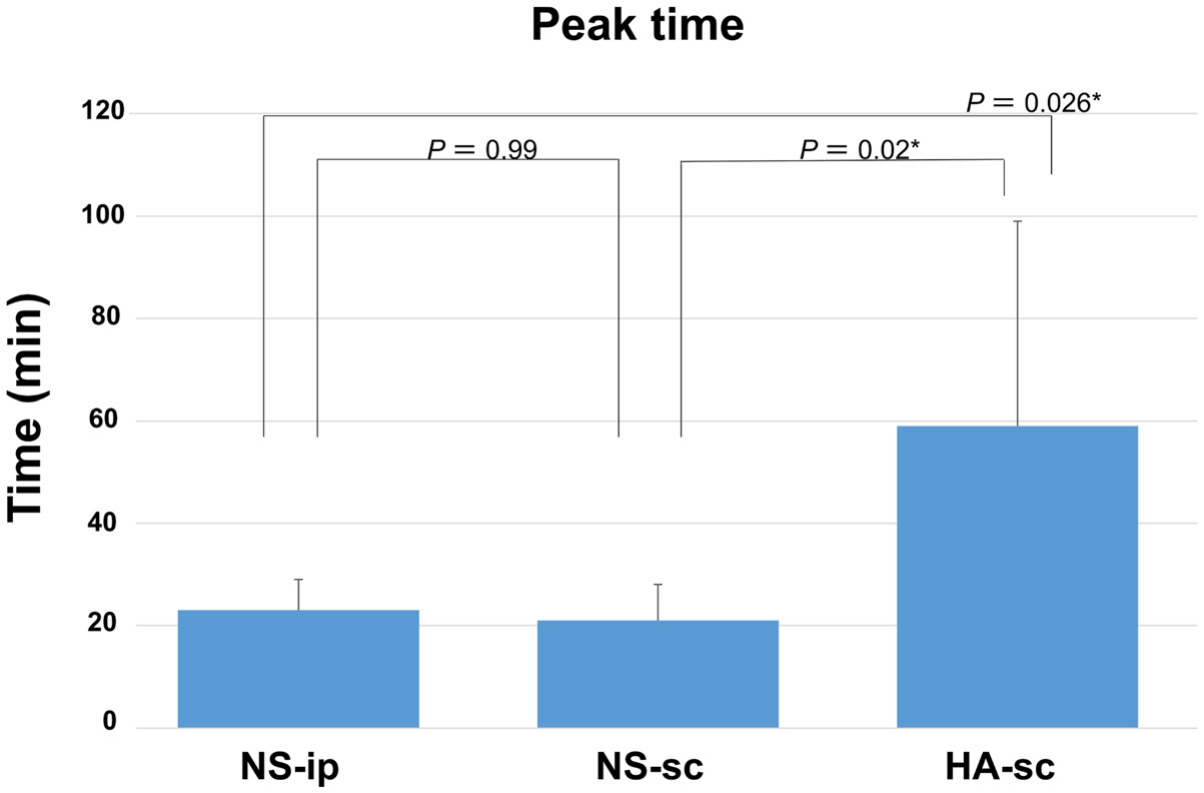
Time at which the peak photon count appeared in each group. The bar graph shows the average value of 7 mice of each group, and the error bar indicates the standard deviation. The asterisks indicate a statistically significant difference.

**Fig 5:**
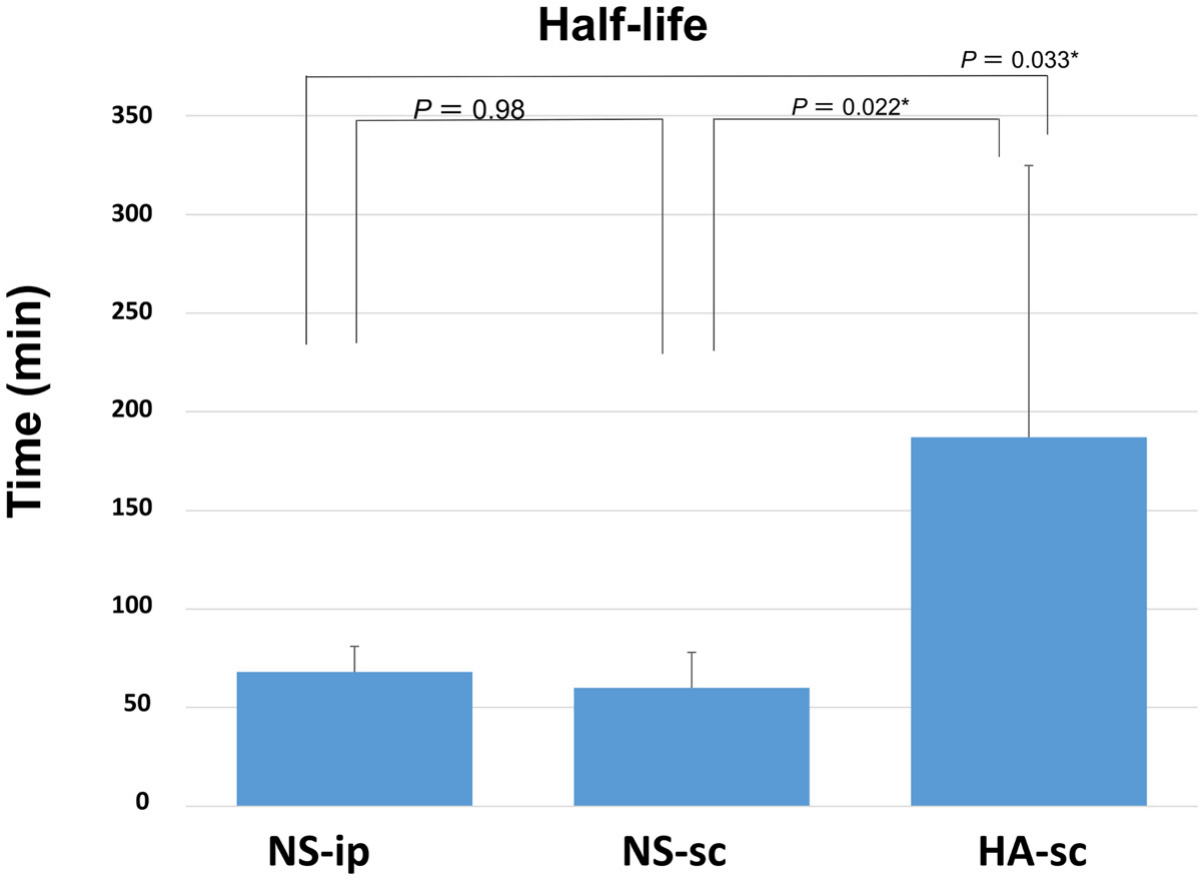
Half-life of fluorescence emission in each group. The bar graph shows the average value of 7 mice in each group, and the error bar indicates the standard deviation. The asterisks indicate a statistically significant difference.

**Fig 6:**
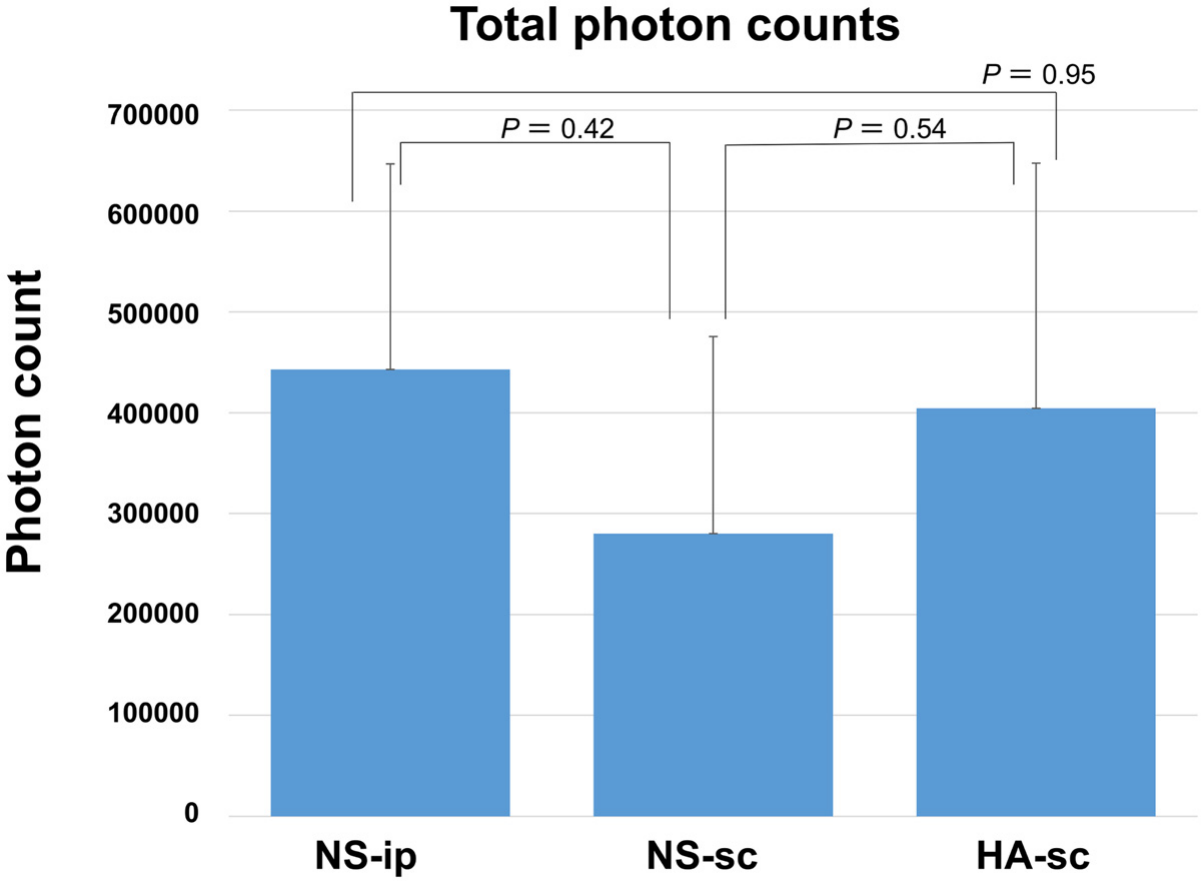
Total photon counts of each group. The bar graph shows the average value of 7 mice in each group, and the error bar indicates the standard deviation.

The maximum peak photon count of the Ns-ip group was reached in 20 min, after which it decreased rapidly. The curve for the NS-sc group was similar to that of the NS-ip group, and the peak value was reached in 20 min. In contrast, the peak value of the HA-sc group was reached in 45 min. The half-life of the HA-sc group was longer than those of the other groups (Figs 1 and 2). An analysis of pharmacokinetic values using the F-test and Tukey’s HSD test did not reveal significant differences in the peak values between any two groups (Fig 3). There were significant differences in peak times between the NS-ip and HA-sc groups and between the NS-sc and HA-sc groups. In both cases, the HA-sc group exhibited significantly longer peak times (*P* = 0.026 and *P* = 0.020, respectively) (Fig 4). Similarly, there were significant differences in the half-lives between the NS-ip and HA-sc groups and between the NS-sc and HA-sc groups. The HA-sc group displayed significantly longer times (*P* = 0.033 and *P* = 0.022, respectively; Fig 5). There were no significant differences in the total photon counts between any 2 groups (Fig 6).

## Discussion

Hearing loss is the most frequent sensory disability, and it may lead directly to a decrease in the quality of life [10, 11]. Moreover, there are few effective treatments [12, 13]. Therefore, advances in the relevant basic research are anticipated by scientists, clinicians, and, of course, people with this condition and their families. Systemic corticosteroids are used to treat acute sensorineural hearing loss. However, its cure rate is an unsatisfactory 30% [14], and the mechanism is unknown. Therefore, it is extremely important to develop robust analytical methods to precisely and easily evaluate the pharmacokinetics of drugs in the inner ear. Further studies are expected to address improving topical drug concentrations. The development of an effective DDS will improve pharmacological effects, decrease side effects, decrease the number of treatments, and improve patients’ quality of life. The blood—inner ear barrier is similar to that of the central nervous system [15, 16].

There are other reports on DDSs targeted to the inner ear [2, 17, 18]. Moreover, there are a few reports on DDSs targeted to the inner ear using hyaluronic acid [13, 19–21]. However, there is no report on the inner ear medicinal dynamics with subcutaneous administration. This the first report on the sustained release effect of subcutaneous administration using hyaluronic acid on the inner ear.

In DDS development, sustained-release formulations are often considered because the concentration in blood of must be maintained at therapeutic levels. In this study, we observed significant increases in the peak time and half-life of luciferin in the luciferin + HA group relative to those in the luciferin + saline groups following subcutaneous injections into the same region. These findings indicate that the HA + luciferin sustained-release formulation significantly suppressed the rapid rise in the blood concentration of luciferin immediately after administration and maintained the concentration of luciferin in the blood over a longer time without HA. Further, HA produced a sustained-release effect of luciferin delivery to the cochlear ganglion.

In fundamental studies, inner-ear pharmacokinetics was studied using dissection and direct measurement of blood concentrations. Therefore, it was impossible to measure changes of inner-ear blood concentrations over time in the same subject. In contrast, our experimental system makes it possible to less invasively monitor the temporal—spatial distribution of changes of a drug in the same individual in vivo. Further, the quantitative data acquired using the IVIS system promise to be useful for determining the pharmacokinetics of drugs targeted to the inner ear. Further, the fluorometric measurements of luciferin oxidation are linear over a wide range of substrate concentrations, and the data presented in this study are an accurate reflection of the pharmacokinetics of luciferin delivered to the spiral ganglion.

When the same amount of the same drug is administered using the same method, the total area under the blood concentration—time curve is constant because of a constant rate of clearance, although the values of the peak and half-life vary. This characteristic explains the lack of significant differences between the administration methods that were independent of the use of a sustained-release preparation.

Conversely, it is known for many sustained-release preparations that it is important to consider the influences of drug metabolism in the target region. Luciferin is degraded by the CYP3A4 isoform of cytochrome P450 [22], and CYP3A4 is present in mouse skin. Therefore, it is highly likely that luciferin was degraded in the region in which the drug was delivered by subcutaneous injection. This finding implies that when a drug persists in a region after its release by a DDS, the drug may be enzymatically degraded. However, in the present study, there were no significant differences in local drug amounts among the 3 groups, a finding that argues against the enzymatic degradation of luciferin.

Moreover, there was no significant difference when luciferin was delivered using the intraperitoneal or subcutaneous route. We expect that a drug will be absorbed from the visceral peritoneum and passed through the portal system, after which it enters the systemic circulation via the liver first-pass effect. Our findings indicate that intraperitoneally introduced luciferin passed through these barriers in the same time required for subcutaneous administration. These pharmacokinetic characteristics are expected to vary depending on the drug. Considering the clinical applications of drugs that treat dysfunction and infections of the inner ear, we expect that the results of preclinical experiments can be generalized to patients.

In the future, we plan to apply our DDS to temporal—spatial analysis of the pharmacokinetics of different drugs administered to the inner ear using multiple doses. Although the present study was limited to cochlear ganglion cells, we plan to construct analytical systems to analyze drug delivery to different cell types to develop innovative treatment strategies that are highly efficient with minimal adverse effects.

## Conclusion

Our imaging system detected differences in the pharmacokinetics of luciferin in the inner ear when it was administered in combination with HA. We demonstrate a sustained-release effect of HA on luciferin, suggesting the promise of HA for controlling drug delivery to the cochlea.
